# Establishment of a secondary infection laboratory model of *Echinococcus shiquicus* metacestode using BALB/c mice and Mongolian jirds (*Meriones unguiculatus*)

**DOI:** 10.1017/S0031182023000604

**Published:** 2023-08

**Authors:** Yantao Wu, Li Li, Fuling Xu, Hongbin Yan, John Asekhaen Ohiolei, Nigus Abebe Shumuye, Xiaofeng Nian, Wenhui Li, Nianzhang Zhang, Baoquan Fu, Wanzhong Jia

**Affiliations:** 1 State Key Laboratory of Animal Disease Control and Prevention/College of Veterinary Medicine, Lanzhou University/National Para-reference Laboratory for Animal Echinococcosis/Key Laboratory of Veterinary Parasitology of Gansu Province/Key Laboratory of Zoonoses of Agriculture Ministry/Lanzhou Veterinary Research Institute, CAAS, Lanzhou 730046, Gansu Province, People's Republic of China; 2 Jiangsu Co-innovation Center for Prevention and Control of Important Animal Infectious Disease, Yangzhou 225009, Jiangsu Province, People's Republic of China

**Keywords:** BALB/c mouse, *Echinococcus shiquicus*, model, Mongolian jird, Protoscolex

## Abstract

*Echinococcus shiquicus* is peculiar to the Qinghai–Tibet plateau of China. Research on this parasite has mainly focused on epidemiological surveys and life cycle studies. So far, limited laboratory studies have been reported. Here, experimental infection of *E. shiquicus* metacestode in BALB/c mice and Mongolian jirds (*Meriones unguiculatus*) was carried out to establish alternative laboratory animal models. Intraperitoneal inoculation of metacestode material containing protoscoleces (PSCs) obtained from infected plateau pikas were conducted on BALB/c mice. Furthermore, metacestode material without PSCs deriving from infected BALB/c mice was intraperitoneally inoculated to Mongolian jirds. Experimental animals were dissected for macroscopic and histopathological examination. The growth of cysts in BALB/c mice was infiltrative, and they invaded the murine entire body. Most of the metacestode cysts were multicystic, but a few were unilocular. The cysts contained sterile vesicles, which had no PSCs. The metacestode materials were able to successfully infect new mice. In the jirds model, *E. shiquicus* cysts were typically formed freely in the peritoneal cavity; the majority of these cysts were free while a small portion adhered loosely to nearby organs. The proportion of fertile cysts was high, and contained many PSCs. The PSCs produced in Mongolian jirds also successfully infected new ones, which confirms that jirds can serve as an alternative experimental intermediate host. In conclusion, a laboratory animal infection was successfully established for *E. shiquicus* using BALB/c mice and Mongolian jirds. These results provide new models for the in-depth study of *Echinococcus* metacestode survival strategy, host interactions and immune escape mechanism.

## Graphical abstract

**Figure:**
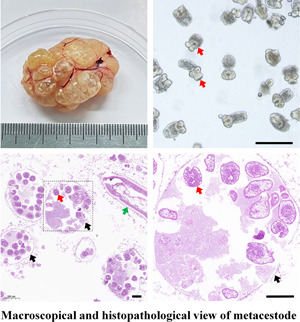

## Introduction

Echinococcosis is one of the deadly zoonotic helminth diseases caused by the larval stages of taeniid cestodes belonging to the genus *Echinococcus* (Wen *et al*., 2019; Wang *et al*., 2021). It not only endangers human health, but also seriously affects the healthy development of animal husbandry, and the economic cost incurred is approximately US$ 3 billion per year (Yu *et al*., 2018; Fu *et al*., 2020). China is one of the countries with the most serious epidemic of echinococcosis, with a threatened population of approximately 60 million and an average prevalence rate of 0.51% (Wu *et al*., 2018; Han *et al*., 2022). In China, Qinghai-Tibet Plateau is a highly endemic area of echinococcosis, and about 80% of cases in China occur in this region (Wu *et al*., 2018; Craig *et al*., 2019). Due to its distinct geological, geomorphic and climatic features, the Qinghai-Tibet Plateau has emerged as one of the most important endemic regions of echinococcosis in the world (Xiao *et al*., 2006; Craig *et al*., 2019). Therefore, the in-depth study of echinococcosis and its pathogen in Qinghai-Tibet Plateau is of great significance for the prevention and control of this disease.

*Echinococcus shiquicus*, as a new species of the genus *Echinococcus* spp. currently limited and endemic to the Qinghai-Tibet plateau region of China, had been mistaken for *E. multilocularis* variant because of its morphologically similar presentation to *E. multilocularis*, and was not identified as a separate species until 2005 based on morphological, molecular genetics, geographical distribution and species evolution characteristics (Xiao *et al*., 2005, 2006; Yan *et al*., 2021). *Echinococcus shiquicus* shares a closer evolutionary phylogenetic relationship with *E. multilocularis*, which makes *E. shiquicus* a sister species to *E. multilocularis* (Nakao *et al*., 2007; Knapp *et al*., 2011). However, the preference for intermediate hosts, the tissue and organ tropism of *Echinococcus* metacestodes and the method of cyst proliferation, differ significantly between these 2 *Echinococcus* species. Meanwhile, there are speculations that the speciation between both of them is a passive host transformation as a result of the evolution of their intermediate hosts, which eventually led to their ecological isolation and gave rise to 2 distinct species (Wang *et al*., 2020; Wu *et al*., 2021). Rodents, for instance, plateau pika (*Ochotona curzoniae*), the plateau vole (*Neodon fuscus*) and carnivorous' mammals, predominantly Tibetan foxes (*Vulpes ferrilata*) serve as intermediate and definitive hosts, respectively (Jiang *et al*., 2012; Li *et al*., 2018, 2019; Han *et al*., 2019). At present, *E. shiquicus* infection has been found in a variety of wild animals such as plateau pika, plateau vole, lacustrine vole (*Microtus limnophilus*), Blyth's mountain vole (*Phaiomys leucurus*) and Tibetan foxes in Sichuan Province, Qinghai Province, Tibet Autonomous Region, Gansu Province and other provinces/autonomous regions in the Qinghai-Tibet Plateau area, China (Boufana *et al*., 2013; Fan *et al*., 2016; Wang *et al*., 2018; Weng *et al*., 2020; Zhu *et al*., 2020). Although there has been no case reports of *E. shiquicu*s infection in humans and livestock to date, the zoonotic potential of this species should not be overlooked (Weng *et al*., 2020; Zhu *et al*., 2020; Yan *et al*., 2021). Currently, our understanding of *E. shiquicus* is mainly based on epidemiological surveys and life cycle studies. So far, laboratory studies have not been reported due to the lack of suitable laboratory animal research model.

Animal model plays a crucial role in the search for novel drugs, immunological patterns and vaccine development (Zhang *et al*., 2017). An ideal experimental model should be based on the natural host of the parasite, but domesticating plateau pika into an experimental animal model is almost an impossible task. Therefore, the establishment of alternative experimental animal models for *E. shiquicus* metacestode would be of great significance in understanding its growth and development, biological characteristics and disease pathogenesis (Zhang *et al*., 2017). The common secondary infection modes of laboratory models of *Echinococcus* include intraperitoneal, intrahepatic, subcutaneous, chest and brain injection of metacestode material. Intraperitoneal inoculation of protoscoleces (PSCs) is easy to operate and easy to observe, and is currently the most widely used animal model of echinococcosis (Romig and Bilger, 1999).

In this study, we focused on establishing a stable and predictable growth pattern animal laboratory model of *E. shiquicus* metacestode by secondary infection of PSCs into the peritoneum of BALB/c mice and Mongolian jirds, small rodent models commonly used in laboratories, which can provide a guarantee for future studies on the biology and developmental biology of *E. shiquicus*.

## Materials and methods

### Experimental animals

Plateau pikas were captured by traps in Shiqu County (Sichuan Province, China) for the collection of cysts from infected lung tissues. Mongolian jirds were purchased from Zhejiang Academy of Medical Sciences and raised in the animal facility of Lanzhou Veterinary Research Institute, Chinese Academy of Agricultural Sciences (CAAS). BALB/c mice (25 g) were obtained from the experimental animal centre of Lanzhou Veterinary Research Institute, CAAS.

### Collection and identification of protoscoleces

Cysts were collected from the lungs of infected plateau pika. Outer cyst surfaces were rinsed with 70% ethanol, and then the cysts were soaked in Dulbecco's Phosphate Buffered Saline (DPBS) containing 100 U mL^−^ penicillin and 100 *μ*g mL^−p^ streptomycin (DPBS-PS) (Gibco, USA), and immediately transported to the lab for treatment. The following procedures were all completed in a sterile setting. The fresh tissues preserved in DPBS were first processed and the cysts were placed in a biosafety cabinet. After 5–6 times of careful washing with DPBS, the cysts were placed in a 60 mm cell culture dish (Corning, USA). Using sterile ophthalmic scissors, cysts were opened to collect the contents mainly including PSCs in 50 mL falcon tubes (Corning, USA). The metacestode suspension was filtered using a 100 *μ*m pore size stainless-steel mesh, thus separating the PSCs from large pieces of metacestode tissue. The flow through was filtered through a 40 *μ*m pore size cell strainer, separating the PSCs from single cells and small cell clumps. The PSCs were then washed off the cell strainer with DPBS, the PSCs in suspension were picked out under a light microscope, and finally the collected PSCs were washed 5 times with DPBS to pellet for 20 minutes (Brehm *et al*., 2003). In the end, the precipitants (PSCs with a few micro-vesicles and fragments) were kept in DPBS-PS. The viability of PSCs was determined by staining with 0.1% methylene blue, with dead PSCs staining blue.

Genomic DNA was extracted from the germinal layer of the cysts according to the manufacturer's instructions of the DNA extraction kit (Qiagen, Germany). Amplification of the mitochondrial *cox*1 gene (471 bp) using forward primer (5′-GCT TTA AGT GCG TGA CTT TTA ATC CC-3′) and reverse primer (5′-CAT CAA AAC CAG CAC TAA TAC TCA-3′) was carried out for all isolates. Positive control was also used (Liu *et al*., 2015). The PCR condition was conducted according to the methods described by Liu et al (Liu *et al*., 2015). Sequences of isolates successfully sequenced (Beijing Tsingke Biotechnology Co., Ltd., Beijing, China) were performed with BLASTN analysis and compared with those previously stored in GenBank. The remaining intact cysts were fixed with 4% paraformaldehyde, embedded in paraffin wax, and sections (4 *μ*m) were stained with hematoxylin and eosin (H&E) and periodic acid-Schiff (PAS), respectively.

### Experimental inoculation of BALB/c mice

PSCs were injected intraperitoneally into 15 BALB/c mice (200 PSCs per mouse). Mice were kept at 20–24°C and 12:12 dark: light photoperiod. The animals were euthanized at 1, 3 and 6 months' post inoculation (p.i.) respectively, and necropsy was carried out. The abdominal cavity was examined for the presence of cysts and their growth and development were recorded. Metacestodes were identified, and cyst contents were collected. Briefly, the cysts were removed and rinsed with DPBS and the outer surface of the cysts was blotted with filter paper. The cysts were punctured, their contents were collected, and whether PSCs exists in the cyst fluid precipitants under light microscopy was observed. Also, the status of PSCs was recorded. Genomic DNA was extracted from cysts' contents for molecular identification. Metacestode material (0.2 mL per mouse) was subsequently serially passaged to new BALB/c mice to maintain the strain. Furthermore, histopathological examination of *E. shiquicus* metacestode was also conducted.

### Experimental inoculation of Mongolian jirds

Subsequently, the metacestode material derived from infected BALB/c mice was transferred by intraperitoneal inoculation into 15 Mongolian jirds (1 ml per jird) aged about 3 months. Those animals were also kept at same environment similar to the mice. The animals were euthanized at 2, 6, 12 months' p.i., and necropsy was carried out. The abdominal cavity was examined for presence or absence of cysts. Metacestodes were separated, and cyst contents were collected. PSCs were observed under microscopic. Genomic DNA was extracted from PSCs for molecular identification as mentioned above. The PSCs (2000 PSCs per jird) were serially passaged to new Mongolian jirds. Histopathologic examination of *E. shiquicus* metacestode was also performed. Total content of cyst containing PSCs were collected. Then its 10 *μ*l were transferred to microscopic slide, and it was covered with cover slip. Number of PSCs was examined and counted under 40 ×  microscope. Finally, the total number of PSCs per experimental animal was calculated.

### Image acquisition and analysis

Bright field and histopathological images were acquired using an inverted microscope (ZEISS, Axio Vert A1) equipped with a CCD camera (ZEISS, Axiocam 305 colour). ZEN software (ZEISS Corporation) and SPSS 12.0 (SPSS Inc.) were employed for image acquisition and statistical data analysis, respectively. Results are presented as the mean ± s.d.

## Results

### Collection and identification of protoscoleces

**Figure 1.**
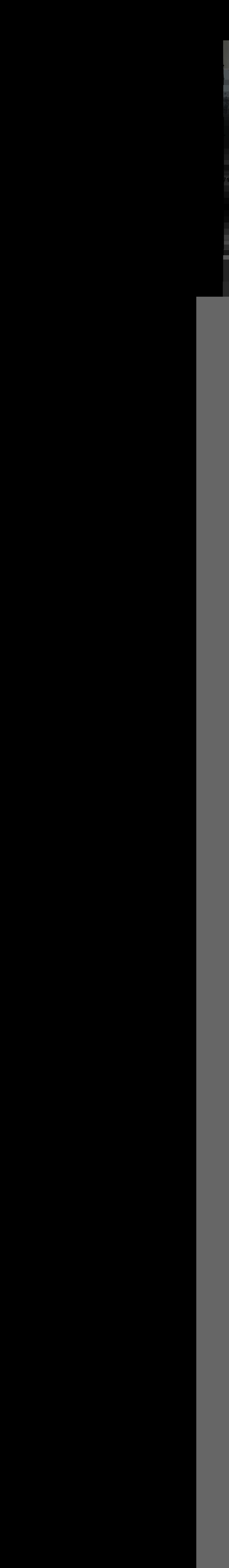
Collection and identification of PSCs of *Echinococcus shiquicus* in plateau pikas. Macroscopical finding in the infected plateau pikas showed the cystic lesions (arrowhead) were all found in the lungs, including single cyst (a), cysts in small groups (b) or cysts in dense aggregations (c), metacestode materials collected in the dish (d), scale bar: 5 mm. Light microscopic of the fertile cysts (e), methylene blue-staining image of PSCs (f) demonstrated that those with no absorbed dye were considered potentially viable (white arrow) and otherwise, they were recorded as dead (yellow arrow). H&E staining of metacestode revealed brood capsules within the cysts (black arrow), and green arrow indicates the germinal layers in the metacestode tissue, scale bar: 100 *μ*m (g). PAS staining of metacestode revealed the laminated layers (blue arrow) (h). Agarose gel electrophoretogram displaying PCR amplified *cox*1 fragments from germinal layer of the cysts. M: Molecular marker 100–2000 bp; Lane 1: test sample; Lane 2, positive control (DNA of *E. shiquicus*); Lane 3, negative control (i).

The cystic lesions of *E. shiquicus* in plateau pikas were enclosed and separated by the internal division of fluid-filled cysts to form multichambered cyst mass, and characterized by either multilocular- or unilocular-cystic structures (Fig. 1a–c). The surface of the cyst was smooth and whitish with clear cystic fluid. The peripheral fibrous layer formed by the host appeared to be thin that is why the cysts were easy to peel off. When transected *in situ*, the laminated layer of the cysts was visually observed to be thin, and the fertile cysts (3–10 mm in diameter sized) were observed overflowing from the metacestode cysts (Fig. 1d). The PSCs were produced individually or in groups either directly from the germinal layer of the cysts (Fig. 1e), and more than 95% of the PSCs were motile (Fig. 1f). Furthermore, most of the PSCs were formed within brood capsulesin the metacestodes shown in the sections stained with H&E (Fig. 1g), and the laminated layers of the cysts were positive for PAS staining (Fig. 1h). Amplification of the *cox*1 gene yielded a PCR product of approximately 471 bp, consistent with the positive control, and subsequent sequencing results indicated that DNA molecules obtained from the germinal layer of the cyst confirmed infection with *E. shiquicus* (Fig. 1i).

### BALB/c mice as alternative experimental intermediate host

**Figure 2.**
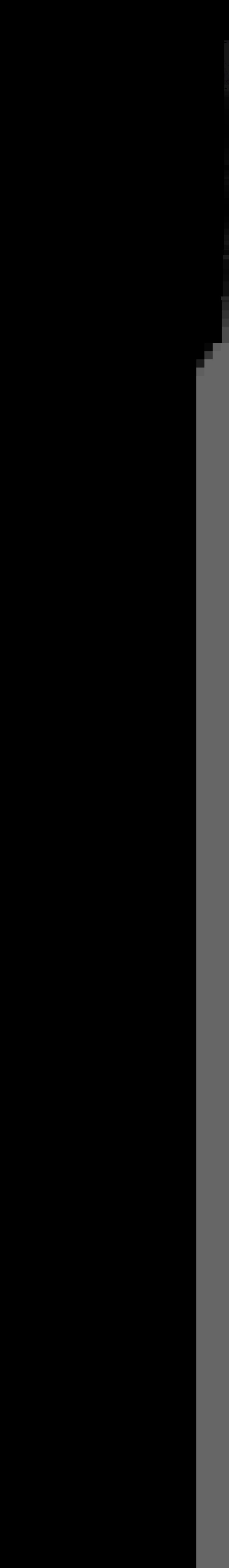
BALB/c mice anatomy after the intraperitoneal injection of *Echinococcus shiquicus* PSCs after 1 (a–e), 3 (f–j) and 6 (k–o) months. After 1 month p.i., cysts lesions (arrowhead) attached to mesentery (a). The contents of the cysts have no PSCs and vesicles (b). After 3 month p.i., cystic lesions infiltrated multiple organs in the abdominal cavity (f). Collected cyst contents were observed to contain a lot of micro-vesicles (red arrow) under light microscopy (g). After 6 month p.i., cystic lesions almost occupied the entire abdominal cavity and organs were severely extruded (k), and micro-vesicles could be observed in the cyst contents (l). H&E staining indicating that metacestode tissue contained sterile cysts (c, h and m). Green arrow indicates the germinal layers of the larvae. Images ‘d’, ‘i’ and ‘n’ are the higher magnification images of the boxed areas in ‘c’, ‘h’ and ‘m’, respectively. PAS staining indicating the laminated layers (blue arrow) of images ‘e’, ‘j’ and ‘o’. Scale bar: 100 *μ*m.

Metacestode of *E. shiquicus* in the mice model frequently attached to adjacent organs. After 1 month p.i., the mice were dissected, and cysts were seen in the mesentery near the small intestine, and the weight of cysts in each mouse was about 0.5 g (Table 1). These cysts contained milky white to yellow porridge-like fluid up on opening the cyst, however, light microscopy and histological investigation failed to detect PSCs in the cyst contents (Fig. 2a–e).

**Table 1.** Overall specific data statistics for all experimental animals

Species of animal	Inoculation time (month)	No. of animals	No. of positives	Average weight (g) of cysts (Σx ± S.D)	Average No. of protoscolices per experimental animal (Σx ± S.D)
BALB/c mice	1	5	3	0.41 ± 0.16	–
	3	5	5	3.78 ± 1.34	–
	6	5	5	7.34 ± 0.84	–
Mongolian jirds	2	5	5	5.67 ± 1.44	–
	6	5	3	19.62 ± 2.39	120 000 ± 2,828
	12	5	5	47.32 ± 4.99	400 000 ± 5,567

At 3 month p.i., cysts grew rapidly, increased several times in size, and began to infiltrate adjacent organs such as liver, spleen and kidney, where the cyst's surface became smooth whitish with clear cystic fluid. The average weight of cysts in each mouse increased over 9 times (3.78 ± 1.34 g) as indicated in the table. Using light microscopy and histopathology, no PSCs were found in the cyst contents, but micro-vesicles were observed (Fig. 2f–j).

**Figure 3.**
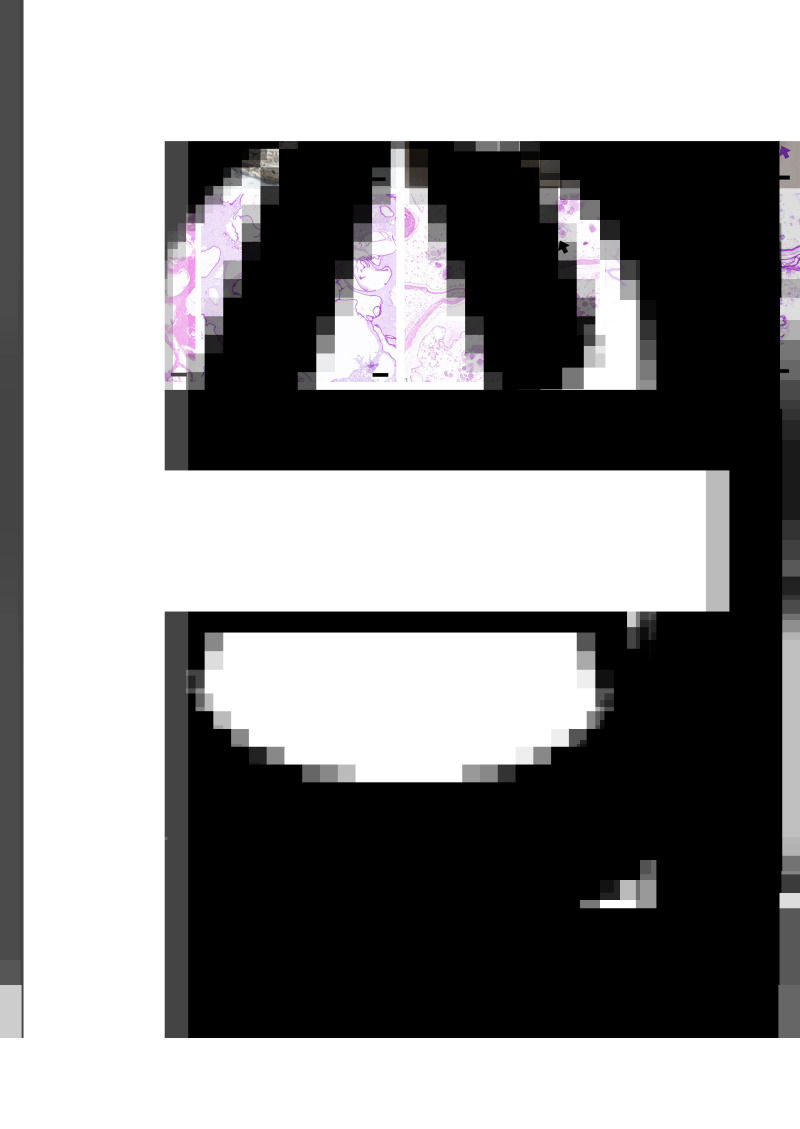
Metacestode tissues of *Echinococcus shiquicus* in the BALB/c mice (a–d) and Mongolian jirds (e–h) 6 month post injection. Black arrow showing cysts observed under light microscope without PSC in mice (a, b), and fertile cysts with PSCs (red arrow) in Mongolian jirds (e, f). Purple arrow showing budding capsule observed under light microscope without PSC in mice (b), and with PSCs in Mongolian jirds (f); Green arrow in the H&E staining showing the germinal layer of the larvae (c, g), and black arrow showing brood capsules with PSCs (red arrow) in Mongolian jirds (g). Blue arrow in the PAS staining showing laminated layer (d, h). Scale bar: 100 *μ*m.

**Figure 4.**
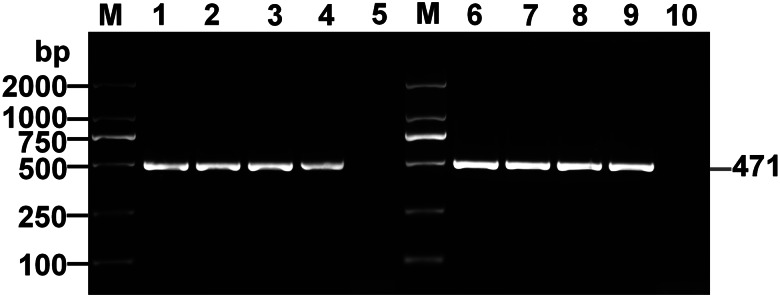
Agarose gel electrophoretogram showing PCR amplified *cox*1 fragments from cysts of BALB/c mice and Mongolian jirds. M: Molecular marker 100–2000 bp; Lanes 1–3, test samples at 1-, 3- and 6-month's post-injection, respectively (BALB/c mice); Lane 4, positive control (*Echinococcus shiquicus*); Lane 5, negative control; Lanes 6–8, test samples at 2-, 6- and 12-month's post-injection, respectively (Mongolian jirds), Lane 9, positive control (*E. shiquicus*); Lane10, negative control.

After 6 month p.i., the cysts invaded the whole body of mice and the average weight of cysts in each mouse were measured to be 7.34 ± 0.84 g (Table 1). Most of the metacestode cysts were multicystic or multilocular while a few were unilocular, and the cysts contained micro-vesicles, but no PSCs were observed (Fig. 2k–o and Fig. 3a–d). Identification of DNA molecules obtained from the germinal layer were successfully amplified (Fig. 4) and sequenced, which confirmed all isolates as *E. shiquicus* up on BLASTN analysis. Overall specific data statistics for all experimental animals are shown in the table. The data implied that the metacestode material containing mainly PSCs of *E. shiquicus* could successfully develop in cysts in mice. And although the weight of cysts increased with the duration of infection, mature PSCs were not observed in our study. On the other hand, the collected metacestode materials were able to successfully infect mice for about 9 generations (data not shown).

### Mongolian jirds as an alternative experimental intermediate host

**Figure 5.**
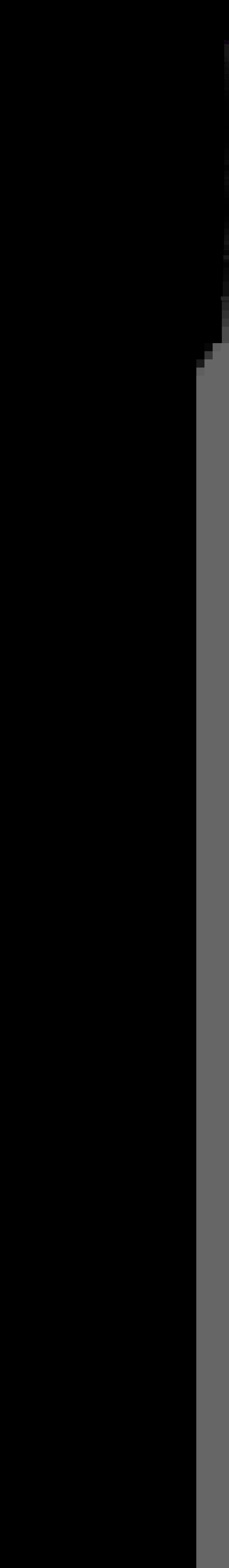
Mongolian jirds anatomy after the intraperitoneal injection of *Echinococcus shiquicus* metacestode materials after 2 (a–f), 6 (g–l) and 12 (m–r) months. After 2 month p.i., cystic lesions (arrowhead) attached to mesentery or free in abdominal cavity (a, b). The contents of metacestode materials had no PSCs and brood capsules (c). H&E staining showed the metacestode had germinal layers (green arrow) (d, e). Blue arrow in the PAS staining showing laminated layer (f). After 6 month p.i., multichambered cystic lesions adhered gently to mesentery but did not infiltrate other organs (g, h). Lots of PSCs (red arrow) in collected cysts contents (i). H&E staining of metacestode revealed fertile cysts within brood capsules (black arrow) (j). Enlarged view of the brood capsule in ‘j’ showed the PSCs in the cysts. Green arrow in the HE staining indicates the germinal layers of the larvae (j, p), and blue arrow in the PAS staining showing laminated layer (l, r). After 12 month p.i., multichambered cystic lesions occupy almost the entire abdominal cavity but did not infiltrate other organs (m, n). H&E staining of metacestode revealed more fertile cysts. Image ‘e’, ‘k’ and ‘q’ is the higher magnification images of the boxed areas in ‘d’, ‘j’ and ‘p’, respectively. Scale bar: 100 *μ*m.

In the Mongolian jirds model, because metacestode grew too slowly during the early phase of infection, data were presented at 2, 6 and 12 months' p.i. *E. shiquicus* cysts were typically developed freely in the peritoneal cavity of Mongolian jirds unlike to those in mice model where they were attached to adjacent organs. Light microscopy and histological investigation at 2 month p.i. revealed that the inoculated metacestode material had begun producing cysts, with an average weight of cysts per jird 5.67 ± 1.44 g (Table 1). PSCs were not observed (Fig. 5a–f).

At 6 month p.i., the development of cysts changed significantly with rapid cyst growth and weight gain, with average weight of cysts per jird being 19.62 ± 2.39 g. Moreover, light microscopy showed the PSCs isolated from the cyst fluid were evaginated or invaginated, and had highly motile. The average number of PSCs in each jird was about 120 000. Histopathological findings demonstrated that the brood capsules appeared as honeycomb-like that were closely arranged, and PSCs were observed in the brood capsules (Fig. 3e–h, 5g–l). Regarding the thickness of the cyst laminated layer, it was thinner in mice than in jird's (Fig. 3d, h). The germinal layer of the cysts also proliferated outwards in a budding manner, and it was the first time that it has been documented that *E. shiquicus* metacestodes can multiply by budding before forming multilocular cysts (Fig. 3b, f).

At 12 month p.i., the cysts were larger in size, and contained a large number of brood capsules and PSCs, with an average weight of cysts increased about 8 times compared to 2 month p.i. (47.32 ± 4.99 g, Table 1). And the average number of PSCs in each jird was about 400 000. However, there was no organ invasion and upon incision. Brood capsules (mean diameter of 300 *μ*m) overflowed from the metacestode (Fig. 5m–r). Similar to the mice model, molecular identification of the isolates implied that they are *E. shiquicus* (Fig. 4). The PSCs collected from Mongolian jirds were able to successfully infect jirds for about 6 generations (data not shown). The overall experiment showed that metacestode material in mice developed into cysts and produced PSCs in Mongolian jirds, and the weight and size of cysts and the number of PSCs increased with the duration of infection.

## Discussion

As a pathogen of potential zoonotic parasitic diseases, *E. shiquicus* infection in humans and livestock has not been reported so far. It is hypothesized that it may be connected to the species' late discovery and recognition, the lack of sufficient experimental materials, etc. (Nakao *et al*., 2013). Several obstacles including the cost of husbandry, management, labour and space availability hinder the establishment of natural intermediate host in the laboratory (Kandil *et al*., 2020). However, the establishment of parasites outside their natural hosts is a significant requirement for understanding their development. Consequently, to better understand *E. shiquicus* and address the potential disease threat in the future, it is important to build a practical, adequate and controllable experimental animal model (Kandil *et al*., 2020). The present study is the first attempt to establish a secondary infection laboratory model of *E. shiquicus* metacestode with commonly used experimental animals.

Due to the low host specificity of *Echinococcus* spp. in the metacestode stage, a wide range of mammal species are known to be natural intermediate hosts, consequently, a long list of species has been used for experimental infections (Breijo *et al*., 1998). The experimental model for secondary echinococcosis established in rodents and lagomorphs by intraperitoneal injection of PSCs into the peritoneum has been proven to be useful in studying basic aspects of *Echinococcus* spp., such as immunobiology, investigating *in vivo* differentiation process of their secondary cysts in a host and the early local interactions between host and parasite during this process, and testing new chemotherapeutic agents or therapeutical protocols, vaccine candidates and diagnostics or follow-up tools (Dempster *et al*., 1991; Breijo *et al*., 1998; Cucher *et al*., 2013; Wang *et al*., 2018; Miles *et al*., 2020). Similarly, infection of laboratory model of *E. shiquicus* metacestode has been successfully established by means of secondary infection through intraperitoneal injection.

Various strains of mice and Mongolian jirds have been used as *E. granulosus* metacestode model hosts. The cysts usually develop free in the peritoneal cavity, but may be attached to or grow into neighbouring organs. Previous report showed that growth is slow in BALB/c mice and most cysts remain sterile even after 15 months of infection (Romig and Bilger, 1999). PSCs can de-differentiate into cysts with laminated layers in white mice after about 1 month, and the germinal layers of cysts can differentiate into PSCs and brood capsules at about 6 months p.i. (Heath, 1970). In Mongolian jirds, cysts grow slowly, and generally do not obtain fertile cysts until 9–12 months p.i. (Thompson, 1976; Garcia-Llamazares *et al*., 1998). Although not all experimental animals can serve as suitable hosts for *E. granulosus*, and the time taken for fertile cysts to develop and yield reasonable numbers of PSCs is considerable. Once the infection is successfully established, sufficient experimental materials can be obtained by serial passage through model animals (Thompson, 1976). Similarly, injection of homogenized metacestode material into the peritoneal cavity is a widely used method in establishing secondary alveolar echinococcosis (AE) in rodents. Meanwhile studies on animal models for *E. multilocularis*, which can produce numerous PSCs, including Mongolian jirds, cotton rats and common voles (*M. arvalis*) have been conducted, where the most rapid growth was demonstrated in Mongolian jirds (Romig and Bilger, 1999). Various strains of mice such as C57L, C57BL/6, AKR, BALB/c and severe combined immunodeficiency mice show considerable differences in metacestode development. BALB/c mouse was considered to be the most suitable host for *E. multilocularis* (Gottstein and Felleisen, 1995). According to previous records of *Echinococcus* infection models, published results from various host species are contradictory in some cases, and the reasons for this result may be related to host specificity, the source and isolate of the parasite material and mode of infection (Romig and Bilger, 1999).

The development of *E. shiquicus* in an attempt to investigate secondary cysts infection through intraperitoneal metacestode material injection in different laboratory animal models shows that mice and jirds are able to support the development of *E. shiquicus* larva despite some differences in cyst characteristics, for example, cysts from mice do not produce PSCs, while cysts produced by jirds can produce large numbers of PSCs (Sato *et al*., 1998; Miles *et al*., 2020). In the present study in Mongolian jirds, as the duration of post infection increases, the size of the cyst as well as the production of PSC increased. The production of PSCs in jirds suggests them as an appropriate model for investigating the growth of secondary cysts. And the 2 animal models of *E. shiquicus* metacestode in the present study will provide sufficient experimental materials for future investigations on the biological characteristics and growth and development of *E. shiquicus*. Infection of different intermediate hosts with the same *Echinococcus* species produces 2 forms of cysts, the fertile and sterile cysts, which may be related to host specificity and immune response to the parasites (Romig and Bilger, 1999; Miles *et al*., 2020). Previous studies have shown that once metacestodes have been established, both fertile and sterile cysts can grow normally as long as the laminated layer remains intact and whether PSCs are produced or not (Hidalgo *et al*., 2019). It was found that metacestode materials from both animal models, whether producing PSCs or not, were able to infect new experimental animals and keep the strain from one generation to the next, possibly because the germinal layer of cysts contains neoblasts, a kind of pluripotent stem cells, which underlie larval development (Brehm and Koziol, 2014; Cheng *et al*., 2019; Kowsari *et al*., 2021).Our results suggested that cysts from mice have a relatively thin laminated layer, which makes it vulnerable to attack by the host immune system resulting in jelly like cystic contents that eventually lead to calcification and then do not produce PSCs (Bortoletti and Ferretti, 1978; Hidalgo *et al*., 2019). Meanwhile cysts in jirds have not only thicker laminated layer but also it has been reported that they lack effective regulatory immune responses as indicated by Romig and Bilger (1999), which could have led to the production of PSCs (Bortoletti and Ferretti, 1978; Romig and Bilger, 1999). Our models will provide sufficient experimental materials for studying host tropism of *E. shiquicus*.

Previous reports have demonstrated that cysts of *E. shiquicus* origin found in the livers of pikas were essentially unilocular, although an oligovesicular cyst has also been reported (Xiao *et al*., 2005). Our present study confirmed similar unilocular feature in plateau pika as well as multicystic metacestodes, so that the disease caused by *E. shiquicu*s may be multicystic echinococcosis. Our established experimental animal model of secondary infection validated this result.

In conclusion, we attempt to develop an alternative experimental animal model for *E. shiquicus* using BALB/c mice and Mongolian jirds by means of secondary intraperitoneal infection. Although, there were significant differences in growth patterns between BALB/c mice and Mongolian jirds models, unilocular, multilocular and multicystic cysts were observed in both experimental models indicating that both experimental animals could be infected by *E. shiquicus*. The established murine model mostly produced sterile cysts unlike the jirds. Whereas, the Mongolian jirds model produced fertile cysts, indicating that they may be more suitable for acting as one kind of experimental animal for *E. shiquicus* metacestode. These animal models not only lay a foundation for maintaining *E. shiquicus* in laboratory conditions, but also provide opportunity to investigate its biological and medical significance, and useful experimental materials for studying the developmental differences of the same parasite in different hosts, parasite-host interaction and drug screening against echinococcosis.

## Data Availability

All the data supporting the conclusion of this article are included in this article.
